# Early Versus Late Anticoagulation for Acute Ischemic Stroke in Atrial Fibrillation: A Systematic Review and Meta-Analysis of 17,380 Patients

**DOI:** 10.3390/neurolint17120198

**Published:** 2025-12-08

**Authors:** Duaa Abdullah Bafail, Abrar Abdullah Bafail

**Affiliations:** 1 Department of Clinical Pharmacology, Faculty of Medicine, King Abdulaziz University, Jeddah 21589, Saudi Arabia; 2 Nuclear Medicine Department, Le Centre Hospitalier Régional Universitaire, 37000 De Tours, France

**Keywords:** ischemic stroke, atrial fibrillation, oral anticoagulants, early anticoagulation, intracranial hemorrhage, systemic embolism, meta-analysis

## Abstract

Background/Objectives: The optimal timing for initiating oral anticoagulants (OACs) after acute ischemic stroke (AIS) in patients with atrial fibrillation (AF) remains uncertain due to potential risks of recurrent stroke and bleeding. This meta-analysis compares early versus late OAC initiation for recurrent ischemic stroke, major bleeding, intracranial hemorrhage (ICH), systemic embolism, and all-cause mortality. Methods: We conducted a meta-analysis of randomized controlled trials (RCTs), prospective, and retrospective observational studies. Data were pooled using random-effects models, and subgroup analyses were performed to assess outcomes by study design. Heterogeneity was quantified using I^2^ statistics. Results: A total of 17 studies were included. Early OAC initiation was associated with a significantly lower risk of recurrent ischemic stroke compared to late initiation (OR = 0.74, 95% CI [0.58, 0.95], *p* = 0.02), with moderate heterogeneity (I^2^ = 36%, *p* = 0.08). No significant difference was observed in ICH rates (OR = 0.74, 95% CI [0.41, 1.33], *p* = 0.32), major bleeding (OR = 1.48, 95% CI [0.51, 4.30], *p* = 0.47), or systemic embolism (OR = 0.65, 95% CI [0.33, 1.25], *p* = 0.20). All-cause mortality showed no difference between early and late initiation (OR = 1.00, 95% CI [0.72, 1.39], *p* = 0.99). Subgroup analyses were consistent with overall findings, and heterogeneity ranged from low to moderate across outcomes. Conclusions: Early initiation of OACs post-AIS in AF patients significantly reduces ischemic stroke recurrence without increasing risks of ICH, major bleeding, systemic embolism, or mortality. These findings support early anticoagulation strategies for selected patients.

## 1. Introduction

Oral anticoagulants (OACs), including both vitamin K antagonists and non-vitamin K antagonist oral anticoagulants (NOACs), are commonly prescribed for secondary prevention of stroke in individuals with non-valvular atrial fibrillation (AF) [1]. Despite their widespread use, the optimal timing for initiating OAC therapy following an acute ischemic stroke in this population remains an area of clinical ambiguity [2]. This uncertainty arises from the need to carefully weigh the benefits of reducing the risk of recurrent ischemic events against the potential for hemorrhagic complications, particularly intracranial bleeding, during the early recovery phase post-stroke [3].

While early administration of OACs might lower the chance of recurrent strokes, it may also elevate the risk of bleeding. Major clinical guidelines, such as those from the American Heart Association/American Stroke Association, the European Society of Cardiology, and the European Stroke Organization, acknowledge the lack of conclusive evidence to support a precise recommendation on when to initiate OACs in this context [4].

A previously conducted systematic review and meta-analysis suggested no significant differences in the rates of recurrent ischemic stroke, intracranial hemorrhage (ICH), or overall mortality between patients who began OAC therapy within the first seven days and those who initiated treatment within two weeks after their ischemic event [5]. However, that analysis primarily relied on observational studies and intra-group comparisons within single-arm meta-analyses, thereby limiting the strength and generalizability of its conclusions. Despite these methodological constraints, the findings implied that both early and delayed initiation strategies may offer similar efficacy and safety profiles.

Recent evidence has been strengthened by three large randomized controlled trials investigating when to begin oral anticoagulation after ischemic stroke in atrial fibrillation. These include the ELAN trial [6], which compared early versus delayed direct oral anticoagulant initiation, the TIMING trial [7], and the AREST trial [8], all designed to clarify safety and efficacy within this clinical context. Collectively, these studies contribute valuable new data that justify a comprehensive update to previous reviews.

Considering these recent findings, we performed a systematic review and meta-analysis to evaluate the relative safety and efficacy of initiating OACs early versus later in patients who experienced an acute ischemic stroke with non-valvular AF. The objective was to support clinical decision making by providing more comprehensive and up-to-date evidence regarding the most appropriate time to begin anticoagulant therapy in this high-risk group.

## 2. Materials and Methods

This systematic review and meta-analysis followed the updated Preferred Reporting Items for Systematic Reviews and Meta-Analyses (PRISMA) guidelines (Supplementary Table S1) [9]. The study protocol was prospectively registered in the International Prospective Register of Systematic Reviews (PROSPERO; registration number CRD420251151476). As the analysis was based exclusively on previously published data, no institutional ethics approval or patient consent was required.

### 2.1. Search Strategy, and Study Selection

A comprehensive literature search was performed across MEDLINE (via PubMed), Cochrane CENTRAL, EMBASE, Web of Science, and Scopus to identify relevant studies. The search terms included “stroke,” “atrial fibrillation,” and “oral anticoagulants.” The search covered all records available from each database’s inception up to 10 January 2025. No language or publication restrictions were applied. To ensure completeness, the reference lists of eligible articles and conference abstracts were also examined manually. Studies such as case reports, case series, editorials, commentaries, and narrative reviews were excluded from consideration. All retrieved publications were screened independently by the reviewers.

### 2.2. Eligibility Criteria

This review included randomized controlled trials (RCTs) and observational cohort studies that examined adult patients diagnosed with acute ischemic stroke and atrial fibrillation. Eligible populations were those who began oral anticoagulant (OAC) therapy shortly after stroke onset compared with those whose treatment was initiated later. To be included, studies had to report at least one of the following outcomes: recurrence of ischemic stroke, intracranial hemorrhage, major bleeding, systemic embolism, or all-cause mortality.

### 2.3. Data Extraction

The extraction of data was performed using structured forms that captured key details such as trial names, patient populations, patient characteristics, types of OACs used, timing of OAC initiation, and reported outcomes.

### 2.4. Outcomes Parameters

The main endpoint analyzed was recurrence of ischemic stroke during follow-up. Additional outcomes included ICH, major bleeding, systemic embolism, and overall mortality within the same follow-up period. Major bleeding was defined according to the criteria of the International Society on Thrombosis and Haemostasis (ISTH), which classify a bleeding episode as major when it results in a hemoglobin reduction of at least 2 g/dL, requires transfusion of two or more units of blood, or occurs at a critical anatomical site. Most of the studies applied the ISTH or an equivalent definition, ensuring consistency in outcome reporting.

### 2.5. Bias of Bias Assessment

The methodological quality of the included studies was evaluated using standardized tools appropriate for each study type. For randomized controlled trials, the Cochrane Risk of Bias 2 (RoB 2) tool was applied [10]. For observational studies, quality appraisal was performed with the Newcastle-Ottawa Scale (NOS) [11]. Each observational study was examined according to three key domains: (a) adequacy of participant selection, (b) comparability of study groups based on demographic and potential confounding factors, and (c) accuracy of outcome or exposure assessment. Two reviewers conducted the evaluations independently, and any discrepancies were resolved through discussion and consensus.

### 2.6. Statistical Analysis

For each dichotomous outcome, odds ratios (ORs) with corresponding 95% confidence intervals (CIs) were derived to compare early versus late initiation of oral anticoagulation after acute ischemic stroke. Subgroup analyses were conducted according to study design, distinguishing randomized controlled trials (RCTs) from prospective and retrospective observational studies. Pooled effect sizes were estimated using a DerSimonian–Laird random-effects model. The degree of heterogeneity across studies was assessed with Cochran’s Q statistic and the I^2^ index. Heterogeneity levels were interpreted as low (<25%), moderate (25–50%), or substantial (>50%). A Q test *p*-value below 0.10 was regarded as evidence of significant heterogeneity. When more than five studies contributed to a specific outcome, publication bias was examined through visual inspection of trim-and-fill funnel plots. Statistical significance for all analyses was defined at a two-tailed *p*-value of less than 0.05. All computations were performed in R software (version 4.4.2) using the “meta” package (https://www.r-project.org/, accessed on 4 October 2025).

### 2.7. Quality of Evidence Evaluation

We evaluated the overall certainty of evidence and the strength of each recommendation using the GRADE framework as described in the Grading of Recommendations Assessment, Development and Evaluation Handbook [12]. This approach assesses domains such as risk of bias, inconsistency, indirectness, imprecision, and publication bias. The cumulative quality of evidence was rated as high, moderate, low, or very low according to GRADE guidance.

## 3. Results

This section summarizes the findings obtained from the included studies. Subsections present detailed outcomes and their interpretation, followed by the conclusions that can be drawn from the analyses.

### 3.1. Literature Search and Study Inclusion

**Figure 1:**
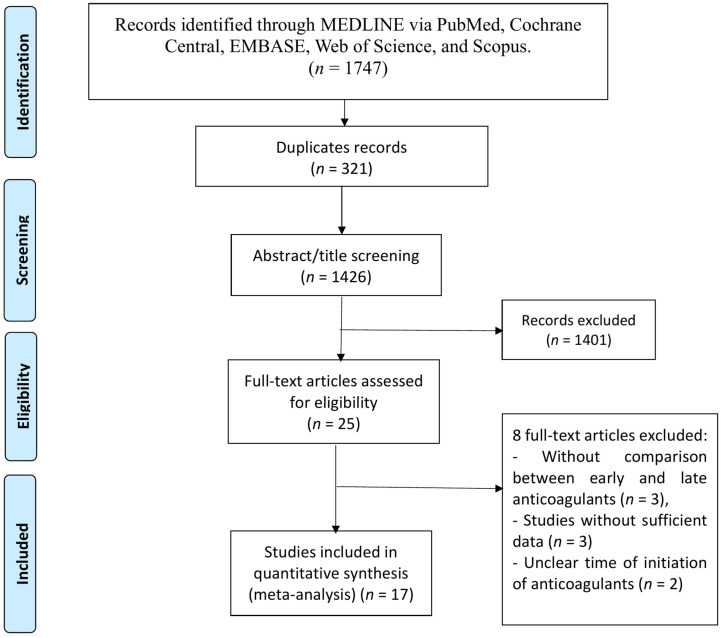
PRISMA flow chart of study selection.

A total of 1426 unique records were identified from the database search. After screening titles and abstracts, 25 studies were selected for full-text evaluation to determine eligibility. Of these, 17 studies satisfied all inclusion criteria and were incorporated into the final meta-analysis. The overall selection process is summarized in the PRISMA flow diagram presented in Figure 1.

### 3.2. Characteristics of Included Studies

We included 17 studies in our analysis, of them, 3 were RCTs [6,7,8], 11 studies [13,14,15,16,17,18,19,20,21,22,23] were prospective observational studies, and 3 [24,25,26] were retrospective observational studies. The 34 included studies investigated a total of 17,380 participants: of them, 9150 received early oral OAC therapy, and 8230 patients received late OAC initiation. The summary of included studies is shown in Table 1.

Across the included studies, baseline stroke severity was comparable between early and late OAC initiation groups. Median or mean NIHSS scores ranged from 4 to 8 in early groups and 5 to 9 in late groups, indicating mild-to-moderate stroke severity. No consistent differences were observed in baseline GCS or ASPECTS scores across cohorts. When reported, the proportion of large-artery infarctions was < 25% in both groups. These data suggest broadly similar baseline neurological severity between groups, supporting the validity of pooled outcome comparisons.

Across the included cohorts, the proportion of patients receiving acute revascularization therapy was modest and comparable between early and late OAC groups. Approximately 15–25% of participants underwent intravenous thrombolysis and 5–10% received endovascular therapy with or without stenting, as reported in the ELAN, TIMING, and AREST trials. Carotid endarterectomy was rare (<2%) and evenly distributed across groups. These interventions were considered during risk-of-bias and sensitivity assessments.

### 3.3. Risk of Bias Assessment of Included Studies

The risk of bias for the included RCTs was evaluated using the RoB2 tool. The included RCTs demonstrated high methodological quality in terms of randomization and outcome reporting. However, they lacked blinding of both patients and investigators. Additionally, the trial by Fischer et al. presented a minor bias due to missing data, while the trial by Oldgren et al. did not specify whether outcome assessments were blinded. Observational cohort studies achieved a moderate quality according to the NOS checklist (Supplementary File S1).

### 3.4. Outcomes Results

#### 3.4.1. Ischemic Stroke Recurrence

**Figure 2:**
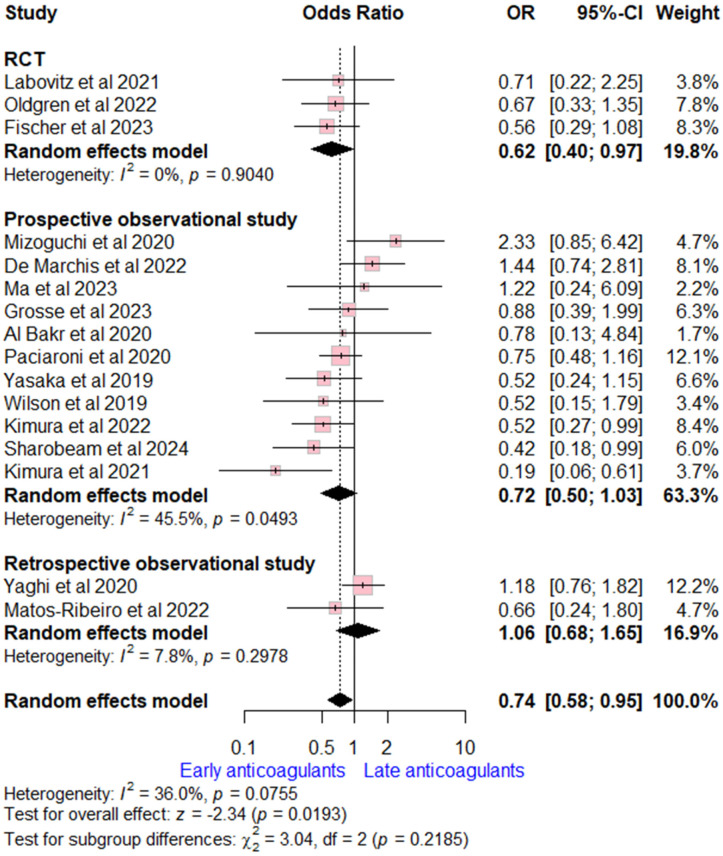
Forest plot comparing early vs. late anticoagulant regarding ischemic stroke recurrence, the solid line marks no effect (OR = 1), while the dashed line indicates the pooled overall effect estimate [6,7,8,13,14,15,16,17,18,19,20,21,22,23,25,26].

Data on recurrent ischemic stroke rates were reported by 16 studies (three RCTs, 11 prospective, and two retrospective observational studies). The pooled analysis revealed early OACs initiation was associated with lower ischemic stork recurrence compared to late OACs (OR = 0.74 (95% CI [0.58, 0.95], *p* = 0.02). Moderate heterogeneity was observed (I^2^ = 0.36, *p* = 0.08). Stratifying by study design, the overall estimate was consistent with RCTs estimate, while no significant difference was found in observational studies (Figure 2).

#### 3.4.2. Intracranial Hemorrhage

**Figure 3:**
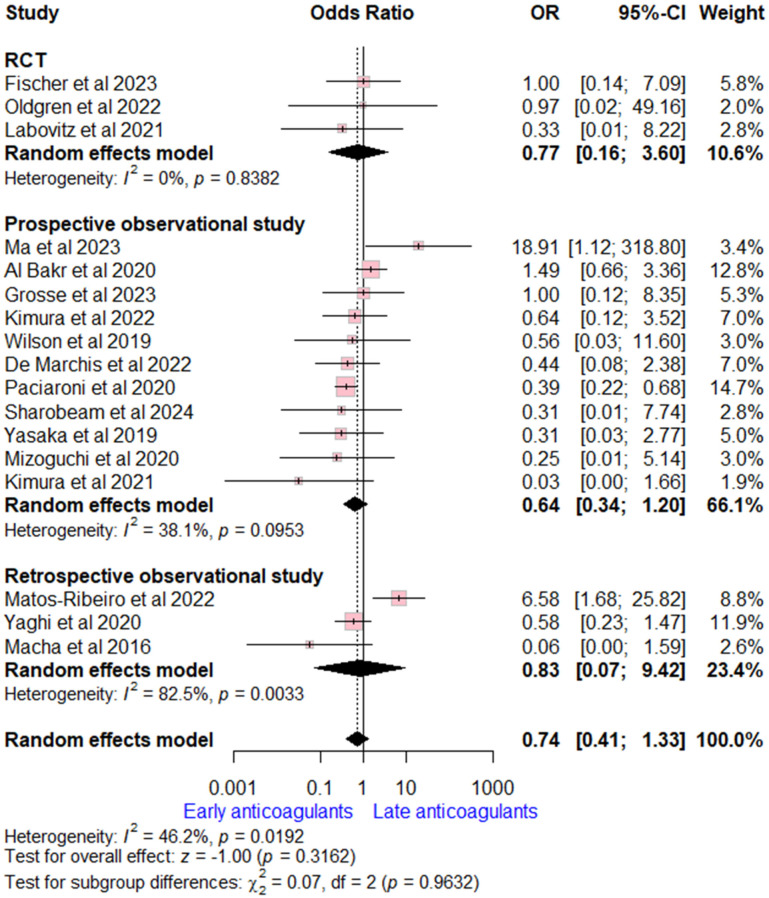
Forest plot comparing early vs. late anticoagulant regarding intracranial hemorrhage, the solid line marks no effect (OR = 1), while the dashed line indicates the pooled overall effect estimate [6,7,8,13,14,15,16,17,18,19,20,21,22,23,24,25,26].

Data on ICH rates were extracted from 17 studies (three RCTs, 11 prospective, and three retrospective observational studies). The overall OR for early versus late OACs was (OR = 0.74, (95% CI [0.41, 1.33], *p* = 0.32), indicating no significant difference. Moderate heterogeneity was detected (I^2^ = 46%, *p* = 0.02). Subgroup analysis according to study design was consistent with the overall estimate (Figure 3).

#### 3.4.3. Major Bleeding

**Figure 4:**
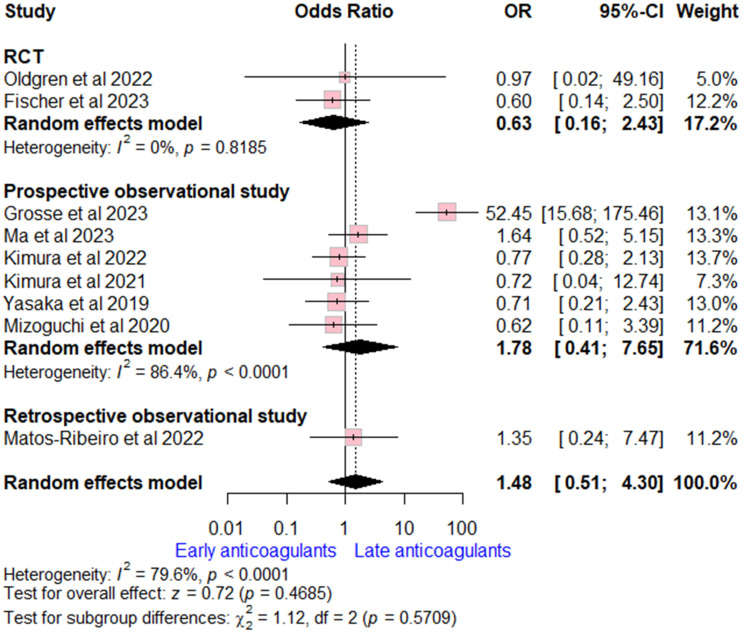
Forest plot comparing early vs. late anticoagulant regarding major bleeding, the solid line marks no effect (OR = 1), while the dashed line indicates the pooled overall effect estimate [6,7,13,14,16,18,19,20,21,22,26].

Data on major bleeding rates were extracted from nine studies (two RCTs, six prospective, and one retrospective observational study). The overall OR for early versus late OACs was (OR = 1.48, (95% CI [0.51, 4.30], *p* = 0.47), indicating no significant difference. High heterogeneity was detected (I^2^ = 80%, *p* < 0.002). Subgroup analysis according to study design was consistent with the overall estimate (Figure 4).

#### 3.4.4. Systemic Embolism

Data on systemic embolism rates were derived from four studies (one RCT and three observational studies). The overall OR demonstrated no significant difference between early and late OACs initiation (OR = 0.65, 95% CI [0.33, 1.25], *p* = 0.20), with moderate heterogeneity observed (I^2^ = 48%, *p* = 0.12). When stratified by study design, the effect size remained consistent in RCTs and observational studies (Supplementary File S2).

#### 3.4.5. All-Cause Mortality

**Figure 5:**
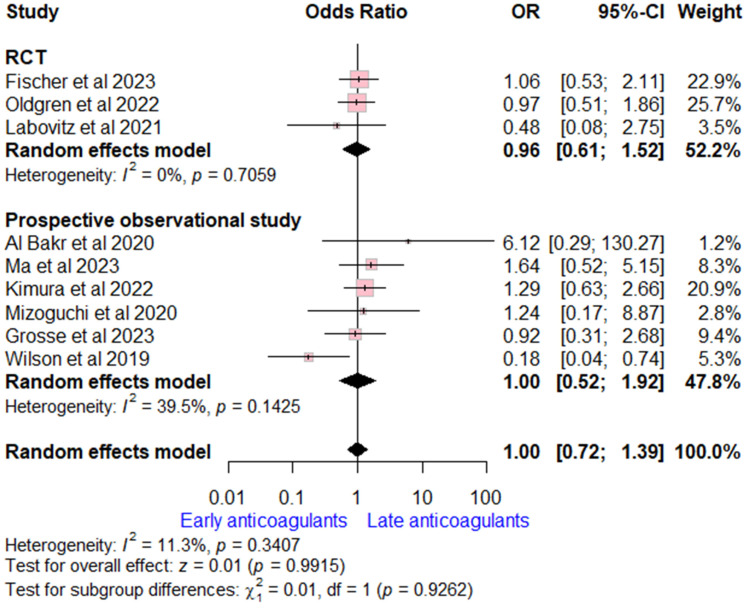
Forest plot comparing early vs. late anticoagulant regarding all-cause mortality, the solid line marks no effect (OR = 1), while the dashed line indicates the pooled overall effect estimate [6,7,8,13,15,16,20,21,22].

Data on all-cause mortality rates were extracted from nine studies (three RCTs and six prospective observational studies). The overall OR for early versus late OACs was (OR = 1.00, (95% CI [0.72, 1.39], *p* = 0.99), indicating no significant difference. No heterogeneity was detected (I^2^ = 11%, *p* = 0.34). Subgroup analysis according to study design was consistent with the overall estimate (Figure 5).

### 3.5. Subgroup and Risk-Stratification Analyses

Exploratory analyses assessed outcomes across clinical-risk strata. When available, studies reported stratification by infarct size, NIHSS, and ASPECTS scores. Pooled data indicated that early OAC initiation was associated with similar benefits in mild-to-moderate strokes (NIHSS ≤ 8) and did not significantly increase bleeding risk in moderate-to-severe cases (NIHSS > 8). No consistent interaction was observed between infarct volume or ASPECTS category and the direction of treatment effect. Patients previously on single antiplatelet therapy comprised roughly 20–30% of total participants and were evenly distributed between groups; exclusion of these patients in sensitivity analyses did not materially alter the pooled estimates. Subgroup comparison of oral-anticoagulant class demonstrated comparable efficacy and safety between vitamin K antagonists (VKAs) and non-vitamin K antagonist oral anticoagulants (NOACs), supporting class-independent findings.

### 3.6. Publication Bias

No significant publication biases were detected for ischemic stroke recurrence, ICH, major bleeding, or all-cause mortality outcomes (Supplementary File S2).

### 3.7. Quality of Evidence Results

According to the GRADE evaluation framework, the certainty of evidence for the analyzed outcomes ranged from moderate to high. None of the assessed endpoints were rated as low or very low in quality. Detailed GRADE assessments are provided in Supplementary Table S2.

## 4. Discussion

This meta-analysis indicates that initiating OACs soon after an acute ischemic stroke in patients with atrial fibrillation is associated with a reduced risk of recurrent ischemic events, while the incidences of ICH, major bleeding, systemic embolism, and all-cause mortality remain comparable between early and delayed initiation groups.

Subgroup evaluations across study designs, including RCTs and both prospective and retrospective observational cohorts, consistently supported the favorable efficacy profile of early OAC initiation without any detectable increase in safety concerns. The evidence overall points toward a trend supporting earlier anticoagulation when clinically appropriate.

The decision regarding optimal OAC timing must still be individualized. Strict adherence to fixed early or late initiation thresholds may oversimplify treatment for this diverse stroke population [27]. For example, patients with a higher risk of bleeding, such as those with hemorrhagic transformation or extensive infarction, may benefit from a delayed approach. However, exploratory analyses from major RCTs, including ELAN and TIMING, did not show significant differences in outcomes according to stroke severity, age, or infarct size [6,7]. Observational evidence, such as that reported by Yaghi et al., also suggests that infarct size or hemorrhagic conversion has little influence on the relationship between OAC timing and clinical outcome [25]. Some registry data indicate a slightly higher risk of ICH in patients with large infarctions, emphasizing the need for individual treatment decisions that consider comorbidities, concurrent medications, and frailty [15].

Previous systematic reviews that included mainly observational data showed similar efficacy and safety outcomes between early and late OAC initiation [5]. With the addition of recently published high-quality randomized studies, the current analysis increases the sample size and strengthens the comparative findings. The results suggest that early OAC initiation is generally safe and may provide improved efficacy in reducing ischemic-stroke recurrence. However, given the moderate heterogeneity and residual confounding across studies, these outcomes warrant cautious interpretation.

Further clarification on the appropriate timing of OAC initiation is expected from ongoing randomized trials, including START (Optimal Delay Time to Initiate Anticoagulation After Ischemic Stroke in Atrial Fibrillation, NCT03021928) and OPTIMAS (Optimal Timing of Anticoagulation After Acute Ischemic Stroke, NCT03759938). These trials will focus on various time windows for NOAC initiation and include previously excluded high-risk patients, such as those with symptomatic ICH or large infarctions. The results of these trials are eagerly anticipated, and they will likely contribute to refining the current understanding of OAC timing.

While our meta-analysis offers valuable insights, certain limitations should be acknowledged. The inclusion of observational studies contributed significantly to the study weights in the analyses, and the lower National Institutes of Health Stroke Scale (NIHSS) scores in patients initiating OACs early could indicate a selection bias in observational studies. Additionally, the studies did not consistently define “early” OAC initiation, but our subgroup analyses showed consistent efficacy and safety regardless of the time window used.

In general, these observations indicate a consistent but not definitive signal favoring early OAC initiation, warranting confirmation in ongoing large-scale randomized trials.

## 5. Conclusions

This updated meta-analysis suggests that early oral-anticoagulant (OAC) initiation in acute ischemic stroke patients with atrial fibrillation appears safe and may provide a modest benefit in reducing ischemic-stroke recurrence. While the findings are consistent across study designs, variations in study definitions, patient selection, and follow-up periods indicate that these results should be interpreted cautiously. Future large-scale randomized trials, such as START and OPTIMAS, will be essential to confirm these trends and refine evidence-based recommendations on the optimal timing of OAC initiation in this high-risk population.

## Figures and Tables

**Table 1:** Baseline characteristics of included studies.

First Author	Year	Study Design	Country	Number of Patients, Early/Late	Age	Time Window of Initiation in Days		Follow-Up	Type of OAC		CHA2DS2-VASc
						Early	Late		VKA	NOAC	
Sharobeam et al. [23]	2024	Prospective observational study	Australia	107/101	74.2	<4 days	≥4 days	3 months	+	+	-
Fischer et al. (ELAN) [6]	2023	RCT	International	1006/1007	77 (70–84)	within 48 h after a minor or moderate stroke or on day 6 or 7 after a major stroke	day 3 or 4 after a minor stroke, day 6 or 7 after a moderate stroke, or day 12, 13, or 14 after a major stroke	3 months	−	+	5
Grosse et al. (PRODAST) [21]	2023	Prospective observational study	Germany	1642/274	76 (55–89)	≤7 days	>7 days	3 months	+	+	5
Ma et al. [22]	2023	Prospective observational study	China	125/66	71 ± 10.15	≤4 days	>4 days	3 months	+	+	4
De Marchis et al. [19]	2022	Prospective observational study	Seven European and Japanese countries	1362/1188	77	≤5 days	>5 days	3 months	−	+	5
Matos-Ribeiro et al. [26]	2022	Retrospective observational study	Portugal	134/100	80 (72–85)	≤4 days	>4 days	3 months	+	+	4
Oldgren et al. (TIMING) [7]	2022	RCT	Sweden	450/438	78.3 ± 9.9	≤4 days	>4 days	3 months	−	+	-
Kimura et al. [20]	2022	Prospective observational study	Japan	785/1012	77 (70–84)	≤4 days	>4 days	3 months	−	+	3
Labovitz et al. [8]	2021	RCT	United states	47/41	73.5 ± 12.7	0–3 days for TIA, 3–5 for small-sized AIS, day 7–9 for medium-sized)	7 days for TIA, 14 days for AIS	6 months	+	+	5
Kimura et al. [18]	2021	Prospective observational study	Japan	191/44	78.1 ± 5.04	≤7 days	>7 days	3 months	+	+	5
Mizoguchi et al. [16]	2020	Prospective observational study	Japan	223/276	75 (69–82)	≤3 days	≥4 days	3 months	−	+	5
Paciaroni et al. [17]	2020	Prospective observational study	International	468/531	77.2 ± 9.5	≤7 days	>7 days	3 months	+	+	-
Yaghi et al. [25]	2020	Retrospective observational study	United States	617/137	76	≤3 days	>3 days	3 months	+	+	4
Al Bakr et al. [15]	2019	Prospective observational study	Saudi Arabia	55/23	64.5 (54.5–72)	≤6 days	>6 days	3 months	+	+	-
Yasaka et al. (RELAXED) [14]	2019	Prospective observational study	Japan	584/721	77.1 ± 9.6	≤2 days	>2 days	3 months	−	+	-
Wilson et al. [13]	2019	Prospective observational	United Kingdom	358/943	76 ± 10	≤4 days	>4 days	3 months	−	+	5
Macha et al. [24]	2016	Retrospective observational study	Germany	41/32	78	<5 days	>6 days	1 month	−	+	4

RCT, randomize controlled trial; OAC, oral anticoagulant; NOAC, new oral anticoagulant; VKA, vitamin K anticoagulant.

## Data Availability

The data that support the findings of this study are available on request from the corresponding author.

## Supplementary Materials

The following supporting information can be downloaded at: https://www.mdpi.com/article/10.3390/neurolint17120198/s1, Supplementary Table S1: PRISMA 2020 Checklist; Supplementary Table S2: Quality of evidence for the included outcomes; Supplementary File S1; Supplementary File S2. Reference [28] is cited in the Supplementary Table S1.

## Abbreviations

The following abbreviations are used in this manuscript:

AF	Atrial Fibrillation
AIS	Acute Ischemic Stroke
AREST	Apixaban for Early Prevention of Recurrent Embolic Stroke and Hemorrhagic Transformation
CI	Confidence Interval
DOACs/NOACs	Direct Oral Anticoagulants/Non–Vitamin K Antagonist Oral Anticoagulants
ELAN	Early versus Late initiation of direct oral Anticoagulants in post-ischemic stroke patients with atrial fibrillatioN
GRADE	Grading of Recommendations Assessment, Development and Evaluation
ICH	Intracranial Hemorrhage
NOS	Newcastle–Ottawa Scale
OAC	Oral Anticoagulant
OR	Odds Ratio
PRISMA	Preferred Reporting Items for Systematic Reviews and Meta-Analyses
RCT	Randomized Controlled Trial
RoB2	Revised Cochrane Risk of Bias Tool for Randomized Trials
START	Optimal delay time to initiate anticoagulation after ischemic stroke in atrial fibrillation
OPTIMAS	OPtimal TIMing of Anticoagulation After Acute Ischemic Stroke
TIMING	Timing of Oral Anticoagulant Therapy in Acute Ischemic Stroke with Atrial Fibrillation
