# Supplementary material for: Early Versus Late Anticoagulation for Acute Ischemic Stroke in Atrial Fibrillation: A Systematic Review and Meta-Analysis of 17,380 Patients
Source: Neurol Int. 2025 Dec 8;17(12):198. doi: 10.3390/neurolint17120198 (PMC12736072; doi:10.3390/neurolint17120198)
Supplement: Supplementary file 1 [file neurolint-17-00198-s001.zip › Supplementary File S1.pdf]

Risk of bias using ROB2 tool.

First author	Year	Randomization style	Deviations from the intended interventions	Missing outcome data	Measurement of the outcome	Selection of the reported results	Overall bias
Fischer et al (ELAN)	2023	+	-	+	+	+	+
Oldgren et al. (TIMING)	2022	+	+	+	+	+	+
Labovitz et al.	2021	-	-	+	+	+	-

Supplementary file 2: Methodological quality of the included observational studies based on the New Castle Ottawa scale for assessing the quality of epidemiological studies.

Study	Year	**Selection**				**Comparability**	**Exposure**			Total Score
		Representativeness of the exposed cohort	Selection of the non-exposed cohort	Ascertainment of exposure[1]	Outcome was not present at start of study[2]	Control for 2 important factors[3.4]	Assessment of outcome	Follow-up long enough	Adequacy of follow-up of cohort[7]	
Sharobeam et al.	2024	*	*	*	*	*	*	*	*	8
Grosse et al. (PRODAST)	2023	*	*	*	*	*	*	*	*	8
Ma et al.	2023	*	*	*	*	*	*	*	*	8

De Marchis et al.	2022	*	*	*	*	*	*	*	*	8
Matos-Ribeiro et al.	2022	*	*	*	-	*	*	*	*	7
Kimura et al.	2022	*	*	*	*	*	*	*	*	8
Kimura et al.	2021	*	*	*	*	*	*	*	*	8
Mizoguchi et al.	2020	*	*	*	*	*	*	*	*	8
Paciaroni et al.	2020	*	*	*	*	*	*	*	*	8
Yaghi et al.	2020	*	*	*	-	*	*	*	*	7
Al Bakr et al.	2019	*	*	*	*	*	*	*	*	8
Yasaka et al. (RELAXED)	2019	*	*	*	*	*	*	*	*	8
Wilson et al.	2019	*	*	*	*	*	*	*	*	8
Macha et al.	2016	*	*	*	-	*	*	*	*	7

[1] If the exposure data was obtained from prescription database or medical record, a point was assigned.

[2] If the study design is prospective study, a point was assigned.

[3] If adjusted for age, a point was assigned.

[4] If adjusted for any other additional factors, a point was assigned.

[5] If the completeness of follow-up was 80% or more, a point was assigned.
