# Supplementary material for: Early Versus Late Anticoagulation for Acute Ischemic Stroke in Atrial Fibrillation: A Systematic Review and Meta-Analysis of 17,380 Patients
Source: Neurol Int. 2025 Dec 8;17(12):198. doi: 10.3390/neurolint17120198 (PMC12736072; doi:10.3390/neurolint17120198)
Supplement: Supplementary file 1 [file neurolint-17-00198-s001.zip › Supplementary File S2.pdf]

Supplementary file

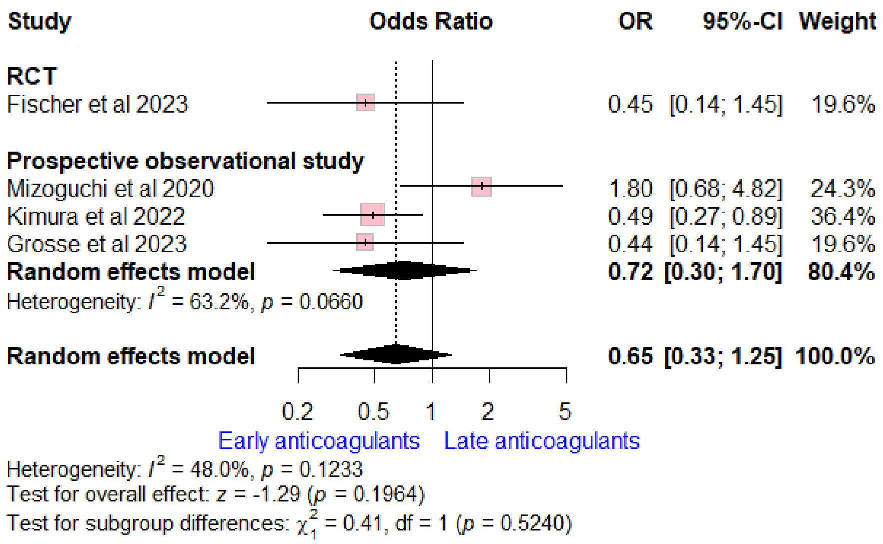

Forest plot comparing early vs late anticoagulants regarding systemic embolism.

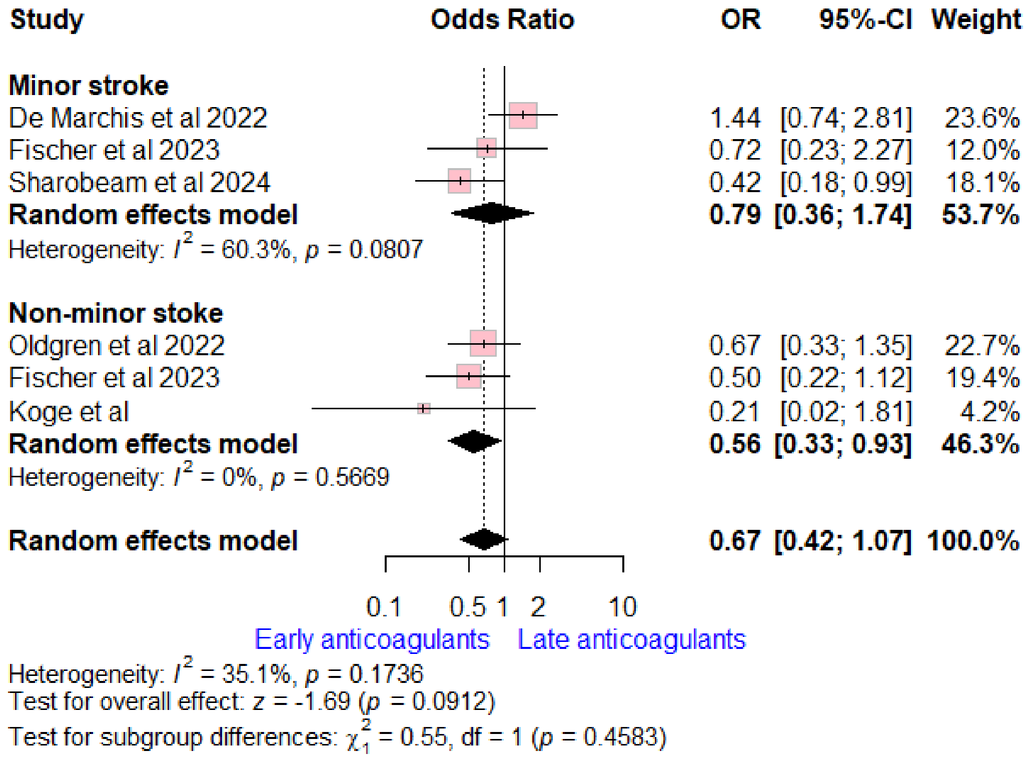

Forest plot of subgroup analysis comparing early vs late anticoagulants regarding recurrent ischemic stroke in minor stoke (NIHSS <5) and non-minor stoke (NIHSS ≥5).

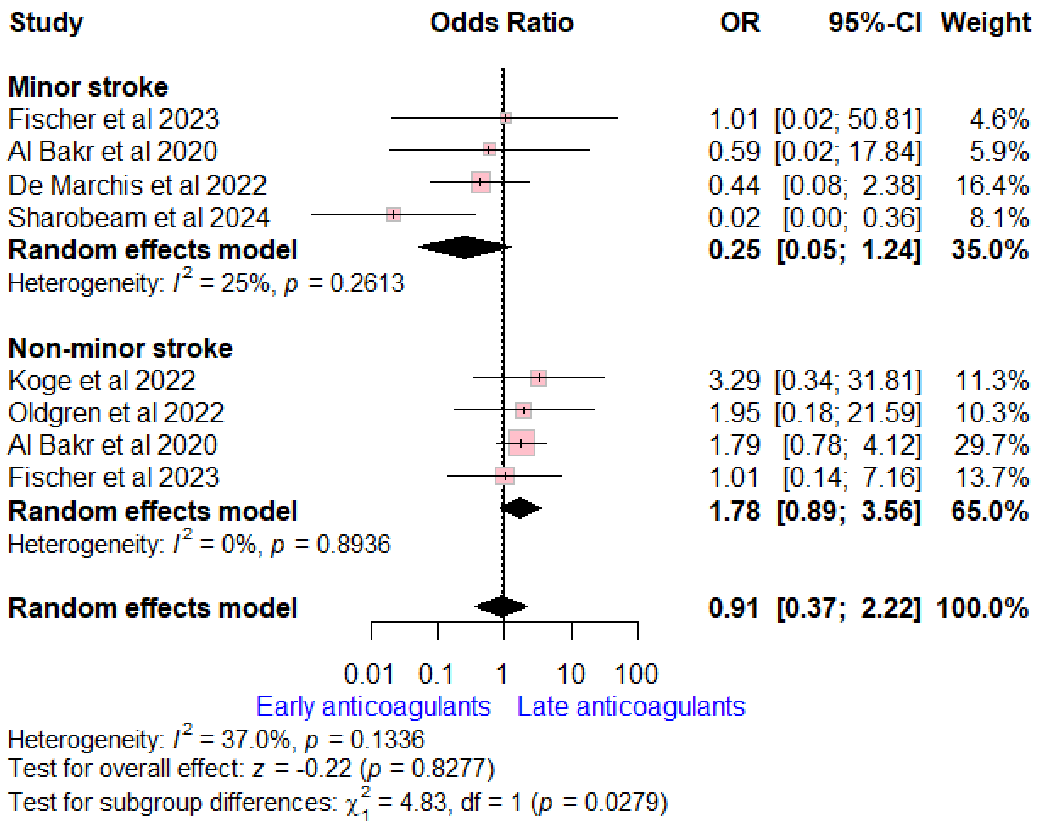

Forest plot of subgroup analysis comparing early vs late anticoagulants regarding intracerebral hemorrge in minor stoke (NIHSS <5) and non-minor stoke (NIHSS ≥5)

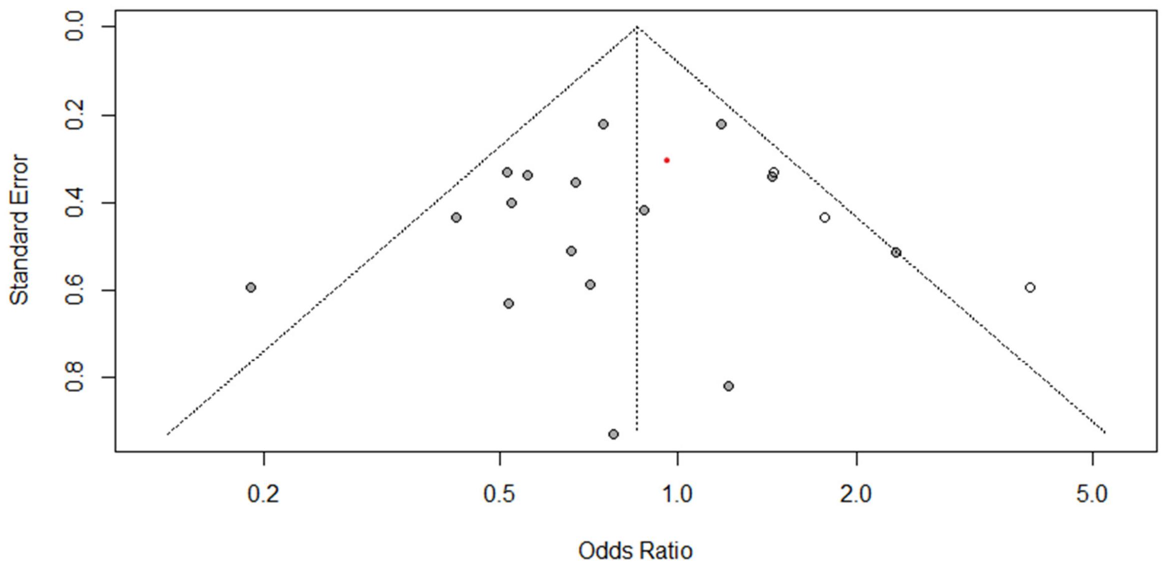

Funnel plot of ischemic stroke

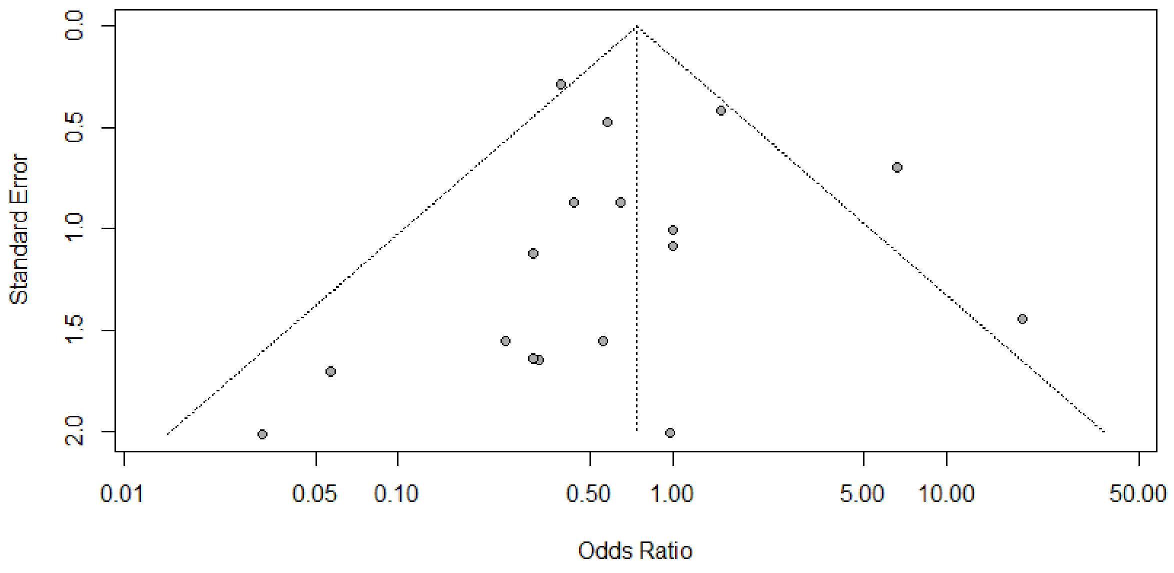

Funnel plot of intracranial hemorrhage

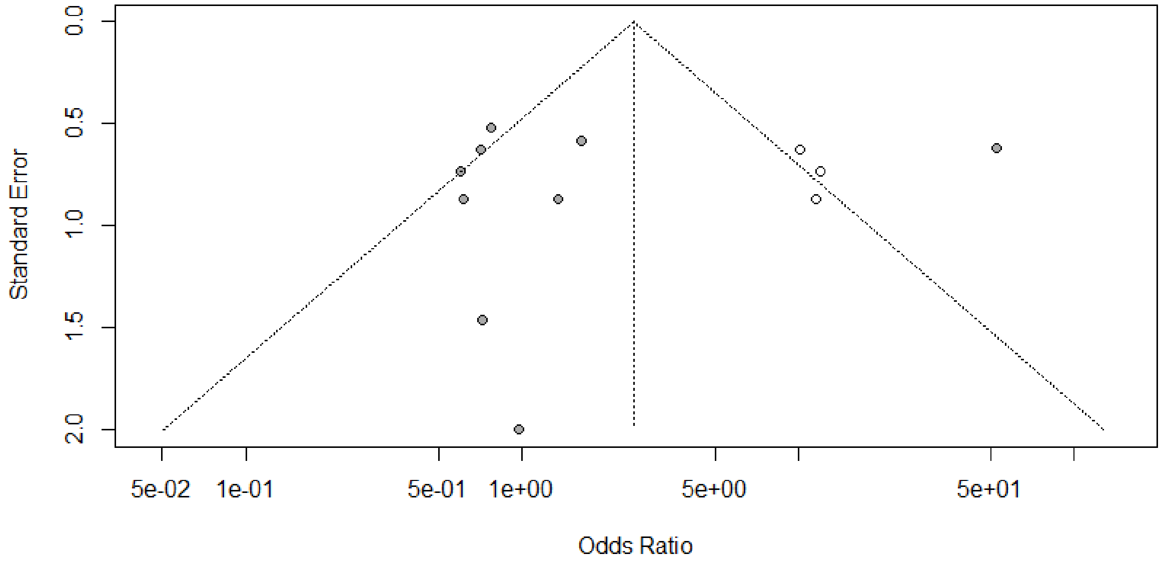

Funnel plot of major bleeding

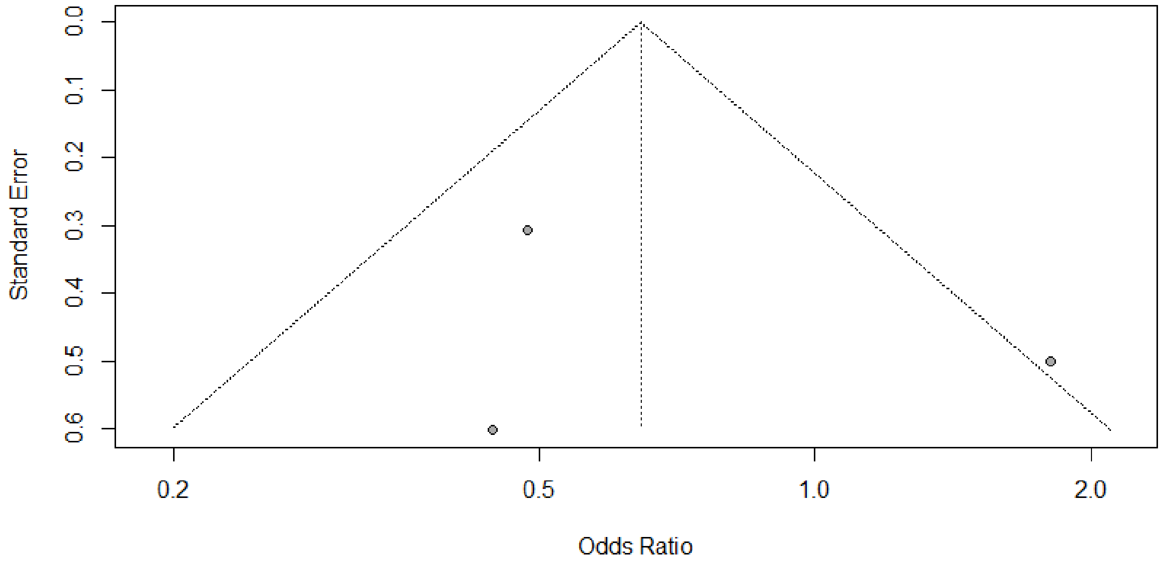

Funnel plot of systemic embolism.

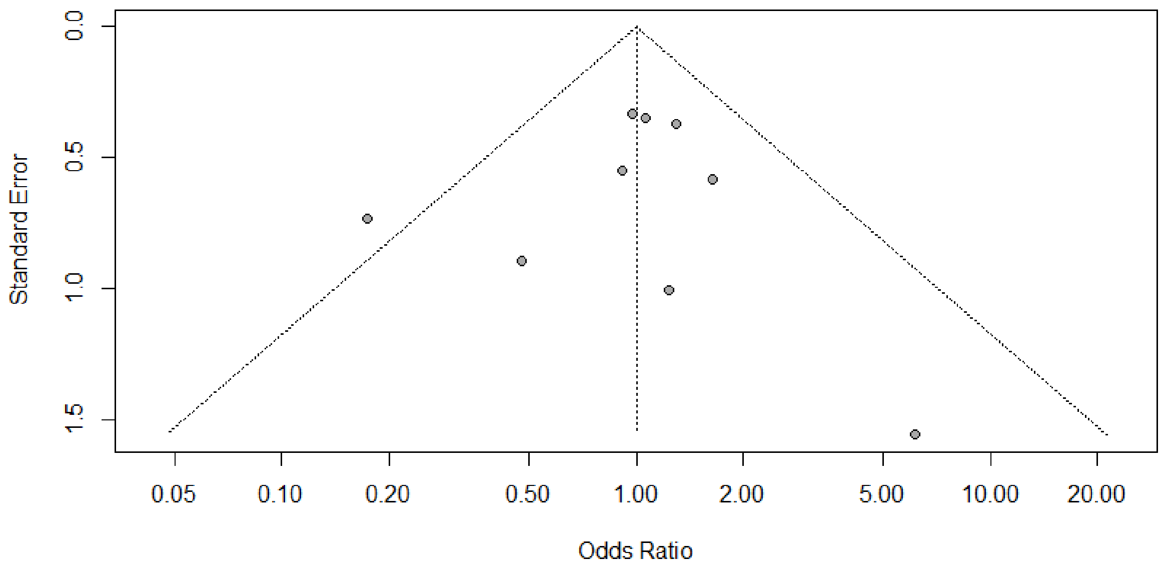

Funnel plot of all-cause mortality
