# Supplementary material for: Early Versus Late Anticoagulation for Acute Ischemic Stroke in Atrial Fibrillation: A Systematic Review and Meta-Analysis of 17,380 Patients
Source: Neurol Int. 2025 Dec 8;17(12):198. doi: 10.3390/neurolint17120198 (PMC12736072; doi:10.3390/neurolint17120198)
Supplement: Supplementary file 1 [file neurolint-17-00198-s001.zip › Supplementary Table S2.pdf]

Supplementary file: Quality of evidence for the included outcomes.

Outcome	Number of studies	GRADE quality assessment					Overall quality of evidence
		Risk of bias	Inconsistency	Indirectness	Imprecision	Publication bias	
Ischemic stroke recurrence	16	Moderate	Series inconsistency (due to significant heterogeneity)	No series indirectness	No series imprecision	Undetected	⊕⊕⊕⊖ Moderate
Intracranial haemorrhage	17	Low	No series inconsistency	No series indirectness	No series imprecision	Undetected	⊕⊕⊕⊕ High
Major bleeding	9	High	No series inconsistency	No series indirectness	No series imprecision	Undetected	⊕⊕⊕⊖ Moderate
Systemic embolism	4	Moderate	Series inconsistency (due to significant heterogeneity)	No series indirectness	No series imprecision	Undetected	⊕⊕⊕⊖ Moderate
Mortality	9	Low	No series inconsistency	No series indirectness	No series imprecision	Undetected	⊕⊕⊕⊕ High
